# Children benefit from morphological relatedness when they learn to spell new words

**DOI:** 10.3389/fpsyg.2013.00696

**Published:** 2013-10-04

**Authors:** Sébastien Pacton, Jean Noël Foulin, Séverine Casalis, Rebecca Treiman

**Affiliations:** ^1^Laboratoire Mémoire et Cognition, Psychology Department, Université Paris Descartes and Institut Universitaire de FranceParis, France; ^2^Psychology Department, Université Bordeaux SegalenBordeaux, France; ^3^Psychology Department, Université Lille IIILille, France; ^4^Psychology Department, Washington UniversitySt. Louis, MO, USA

**Keywords:** spelling, morphology, implicit learning, self-teaching, spelling acquisition

## Abstract

Use of morphologically related words often helps in selecting among spellings of sounds in French. For instance, final /wa/ may be spelled *oi* (e.g., *envoi* “sendoff”), *oit* (e.g., *exploit* “exploit”), *ois* (e.g., siamois, “siamese”), or *oie* (e.g., *joie* “joy”). The morphologically complex word *exploiter* “to exploit”, with a pronounced *t*, can be used to indicate that the stem *exploit* is spelled with a silent *t.* We asked whether 8-year-old children benefited from such cues to learn new spellings. Children read silently stories which included two target nonwords, one presented in an opaque condition and the other in a morphological condition. In the opaque condition, the sentence provided semantic information (e.g., *a vensois is a musical instrument*) but no morphological information that could justify the spelling of the target word's final sound. Such justification was available in the morphological condition (e.g., *the vensoisist plays the vensois instrument*, which justifies that *vensois* includes a final silent *s*). 30 min after having read the stories, children's orthographic learning was assessed by asking them to choose the correct spelling of each nonword from among three phonologically plausible alternatives (e.g., *vensois*, *vensoit*, *vensoie*). Children chose correct spellings more often in the morphological condition than the opaque condition, even though the root (*vensois*) had been presented equally often in both conditions. That is, children benefited from information about the spelling of the morphologically complex word to learn the spelling of the stem.

## Introduction

Written systems such as English and French represent information about both phonemes (the minimal units of sound in language) and morphemes (the minimal units of meaning). For example, the word *replaced* is made up of seven phonemes and three morphemes (*re* + place + ed). The alphabetic basis of these writing systems explains the crucial role of phonological awareness and knowledge of phoneme–grapheme correspondences in the acquisition of the systems (e.g., Bruck and Treiman, 1990; Bosman and Van Orden, 1997; Sprenger-Charolles et al., 2003). However, use of phoneme–grapheme correspondences does not permit correct spelling of many words in English (Ziegler et al., 1997) and French (Véronis, 1988; Ziegler et al., 1996). Because these writing systems also represent meaning-based aspects of the language, use of morphological relationships between words could help people to achieve the correct spellings of many words (e.g., Pacton and Deacon, 2008).

Many morphological effects on spelling are captured by the principle of root consistency, which states that the roots of words often retain their spelling in related words. The three following examples show how the use of this principle can help spellers to choose among plausible spellings, to spell words for which preservation of regularities at the level of morphemes violates those based on phonemes, and to represent aspects of the written language that have no phonological counterpart. In English, use of the root *heal* can help in spelling the morphologically related word *health* with *ea* rather than with *e* as in *bed* or *ai* as in *said.* In French, the sound /\textschwa/ is typically spelled with *e* (e.g., *retard* “late”; *jeter* “to throw”), but it is spelled *ai* in *faiseur* (/fəzøer/ “producer”) to preserve its morphological origins (it is derived from *faire* /fε r/ “to do”). Also in French, many words end with a silent letter that is motivated by morphology. For example, the masculine adjective *bavard* (/bavar/ “a talkative man”) and the noun *chant* (∫ã/ “song”) have final letters (*d* and *t*, respectively) that are not heard. The decision to include a consonant letter at the end of these words at all and the selection of one specific consonant can often be made by reference to the root word's ending. For example, the feminine adjective *bavarde* (/bavard/ “a talkative woman”) and the verb *bavarder* (/bavarde/ “to chat”) guide spellers to use *d* at the end of *bavard*, and the infinitive verb *chanter* (/∫ãte/ “to sing”) guides spellers to use *t* at the end of *chant*.

Although it takes children a long time to learn to spell some morphemes correctly, for example the inflectional endings in English (Nunes et al., 1997) and French (Totereau et al., 1997), children seem to be sensitive to certain morphological dimensions from an early age (e.g., Treiman and Cassar, 1997). This evidence largely comes from studies showing that misspellings are less common when spellers can use morphological information than when they cannot (Rubin, 1988; Treiman et al., 1994; Treiman and Cassar, 1996; Kemp, 2006). For example, Treiman et al. (1994) studied how children spelled the flap, a sound in American English that occurs between vowels and that is pronounced more like /d/ than /t/. They contrasted children's spelling of flaps in two-morpheme words such as *dirty* and one-morpheme words such as *duty*. The spelling of the flap can be elucidated by referring to the roots of the two-morpheme words (e.g., *dirt* in *dirty*), but there is no such root for the one-morpheme words. Five- to eight-year-old children were more likely to spell the flap correctly in two- than in one-morpheme words. This result suggests that even young children have some ability to use morphological relationships to aid their spelling.

Using a similar logic, Kemp (2006) examined how 7-year-old children spelled medial /z/ in one- and two-morpheme words. There are several alternative spellings for /z/ in this position, including *s*, *z*, and *zz*. For two-morpheme words, the choice between them can be determined by the spelling of the root (e.g., *noisy* is derived from *noise*, but the one-morpheme word *busy* has no such root). The children were more accurate in their rendition of /z/ in two- than one-morpheme words, suggesting that they used morphologically related words to clarify the spelling of the sound.

The studies reviewed so far involve learners of English, but French children also appear to use morphologically related words to help spell other words (e.g., Sénéchal, 2000; Sénéchal et al., 2006; Pacton et al., 2007; Casalis et al., 2011). Sénéchal (2000) asked 7- and 9-year-old Francophone Canadian children to spell three types of words. Some of the words, called morphological words, had a derived form that indicated how to spell the word-final silent consonant. For example, *grand* (/grã/ masculine adjective “tall”) and *camp* (/kã/ “camp”) end in a silent consonant that is pronounced in derived forms such as *grande* (/grãd/ feminine adjective “tall”) and *camper* (/kãpe/ “to camp”). The experiment also included opaque words such as *jument* (/ʒymã/ “mare”), which have no such related word. Finally, the experiment included words like *tiroir* (/tirw
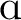
r/ “drawer”), which did not have a silent final consonant. The children were most accurate in their spelling of the words that did not include a final silent consonant. Nevertheless, they were more likely to be correct on the morphological words than the opaque words. This finding suggests that children referred to related words in determining the spelling of the silent endings.

Once French children have learned that many words include a final silent consonant, they sometimes add a silent consonant erroneously to words that do not include such letters. However, reference to morphologically related words such as the verb *citronner* (/sitrone/ “to add lemon to something”) can help in deciding that the noun *citron* (/sitr\textopeno/ “lemon”) is spelled with *on* rather than with a silent final *d* or *t*. Pacton et al. (2007) found that 8- to 10-year-old children incorrectly added a final silent letter to words such as *caleçon* (/kals\textopeno/ “boxer shorts”), which do not have any morphologically related words suggesting the absence of a final silent letter, more often than to words such as *citron*, which have morphologically related words suggesting this absence.

Although the evidence so far suggests that even young children use morphologically related words to spell other words, many of the results are subject to an alternative explanation. Specifically, it is possible that the observed differences between words that have morphologically related words and those that do not reflects a difference in the frequency of the target segments within words. Even if specific words are balanced for their frequency, target segments are typically more frequent in the words that have morphologically related words than in the words that do not. For example, even if *dirty* and *duty* have the same frequency, the segment *dirt* is more frequent than the segment *dut* because *dirt* occurs in words such as *dirty* and *dirtiness* while *dut* occurs only in *duty*.

Deacon and Bryant (2005a,b, 2006a,b) addressed this potential alternative explanation by contrasting spelling of two- and one-morpheme words that begin with the same letter-sound sequence (e.g., *rock* in *rocked* and *rocket*) rather than with different ones (e.g., *dirt* in *dirty* and *dut* in *duty*). For example, Deacon and Bryant (2005a) asked 6- to 8-year-old children to spell the initial segments of two-morpheme words (e.g., fill in the missing letters in ____ed for *rocked*) and their one-morpheme counterparts (e.g., fill in the missing letters in ____et for *rocket*). Children performed better in the former case than the latter, even though they spelled the same sequence in both cases. According to Deacon and Bryant (e.g., 2005a), such findings suggest that children's better performance on two-morpheme words than one-morpheme words does not reflect differences in the orthographic frequency of word forms since the comparison involved the same target segments in both cases. These conclusions were, however, grounded in results obtained in specific and rather artificial conditions. In particular, the morphemic division is already performed when children are asked to fill in the missing letters in ___ed for *rocked*. This is also true in experiments in which children were provided clue words such as *payment* (two-morpheme word) or *pigment* (one-morpheme word) to spell derived words such as *pavement* (Deacon and Bryant, 2005a).

The aim of the present study was to investigate whether children benefit from morphological relatedness in a situation in which such a benefit cannot be explained by a difference in the frequency of orthographic forms between words that have morphologically related words and those that do not. We did so by examining the learning of novel spellings. The use of nonwords allowed us to control strictly the number of presentations of the items and their constituent elements. To make the situation more natural than the one investigated by Deacon and Bryant (e.g., Deacon and Bryant, 2005a), we had children read stories in which the nonwords were embedded as if they were real words. This procedure has previously been used to explore orthographic learning (e.g., Share, 1999; Nation et al., 2007), and it captures the everyday situation in which children encounter new words while reading and then later try to remember their spellings. Different than in other studies using this procedure, some nonwords had morphologically related nonwords that justified the spelling of the final sound and others did not. We refer to these two conditions as the morphological and the opaque conditions, respectively. Our main question was whether, in a spelling test given after the stories had been read, children would perform better on nonwords that had been presented in the morphological condition than on nonwords that had been presented in the opaque condition.

The nature of the two conditions can be made clear with examples. In the opaque condition, for example, children read that, among musical instruments, the *vensois* was probably the hardest to learn and that the musician played the *vensois*. Some children read a version of the passage with the spelling *vensois* and others read a version with *vensoit*. According to French spelling-to-sound rules, both of these are pronounced with a final vowel, as /wa/. Nothing in the passage justified whether the final silent letter of the spelling was *s* or *t*. The corresponding story in the morphological condition further indicated that the *vensoisiste* (*vensoitiste*) was the musician who plays the *vensois* (*vensoit*) and that the *vensoise* (*vensoite*) was a nice melody that can be played only on a *vensois* (*vensoit*). In the related words *vensoisiste* (*vensoitiste*) and *vensoise* (*vensoite*), the *s* (or *t*) is not silent. Thus, in the same way that morphologically related words such as *bavarder* (“to chat”) and *bavardage* (“chatting”) justify the final silent *d* of the word *bavard*, the nonwords *vensoisiste* and *vensoise* justify the final silent *s* of *vensois.* Likewise, *vensoitiste* and *vensoite* justify the final silent *t* of *vensoit*.

The nonword of interest (e.g., *vensois*) was presented seven times in the story in the opaque condition. In the morphological condition, the nonword was presented five times and each of two morphologically related nonwords (e.g., *vensoisiste*, *vensoise*) was presented once. With this procedure, in which the specific nonwords of interest were presented more frequently in the opaque condition than the morphological condition and the target segment was presented the same number of times in the two conditions, better learning of the morphological nonwords than the opaque nonwords could not be explained by a difference of frequency of the orthographic forms.

If children benefit from morphologically related words in spelling, then they should perform better on the final spelling test—in which they were asked to choose the correct spelling of each nonword from among three phonologically plausible alternatives—in the morphological condition than the opaque condition. If the results of previous studies reflect differences in the frequencies of specific spelling sequences, as outlined previously, or if the results are limited to specific and rather artificial situations, then we may not find a difference between the two conditions.

We tested children in Grade 3 (around age 8) because most children at this level are good at applying phoneme–grapheme correspondence rules and because many studies using real word spelling tasks have reported that correct spellings are more common when morphology can be used than when it cannot (e.g., Sénéchal, 2000; Sénéchal et al., 2006) from grade 3 onward, even though the benefit of morphological relatedness has also been evidenced earlier in some studies. As is typically the case in French schools, the children in the present study had not received systematic explicit teaching concerning the principle of root consistency.

## Methods

### Participants

The participants were 28 third graders (13 females), with a mean age of 8.67 years (*SD* = 0.30), from a French primary school located in an area of average socio-economic status in Bordeaux, France. They were tested in the middle of the school year. The children all spoke French as their native language and had no language problems according to their teachers. Their average general spelling ability score, assessed with the Corbeau standardized spelling subtest (Chevrier-Muller et al., 1997) was 38.64 out of 50 (*SD* = 6.45). The average score on this test is reported to be 32.38 for third graders, meaning that the spelling ability of the children involved in this study was somewhat above the expected level of French third graders.

### Stimuli

#### Nonwords

We constructed 16 bisyllabic target nonwords that were phonologically legal in French. The pronunciation of each nonword ended with one of four sounds. As Appendix A shows, for each participant, each target sound was spelled in one way in two nonwords and in another way in two others (e.g., *oit* in *modoit* and *vensoit; ois* in *lagois* and *ridois*). Each spelling was used in one nonword in each condition. Two parallel sets were created to increase the generalizability of findings beyond a single nonword list and to ensure that the results do not reflect one spelling sequence being preferred to another. For example, *modoit* was the target nonword in the opaque condition and *vensoit* was the target nonword in the morphological condition in Set A while *modois* was the target nonword in the opaque condition and *vensois* was the target nonword in the morphological condition in Set B. Conversely, *lagois* was the target nonword in the opaque condition and *ridois* was the target nonword in the morphological condition in Set A while *lagoit* was the target nonword in the opaque condition and *ridoit* was the target nonword in the morphological condition in Set B.

For each target nonword presented in the morphological condition, two morphologically related nonwords were constructed, for example *vensoise* (*vensoite*) and *vensoisiste* (*vensoitiste)* for *vensois* (*vensoit*) and *mansine* (*mansaine*) and *mansinage* (*mansainage*) for *mansin* (*mansain*). Morphologically related nonwords justified the spelling of the final sound of the target nonword. For example, *vensois* includes a final silent *s* that is pronounced in *vensoise* and *vensois*iste and /mãs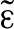/ is spelled with *in* rather than with *ain* because *mansine* and *mansinage* are spelled with *in* rather than with *ain*.

#### Stories

We created eight stories, a sample of which appears in Appendix B. The average length was 157 words (range 153–167). Each story included one target nonword in the opaque condition, one target nonword in the morphological condition, and two morphologically related nonwords. In each story, the two target nonwords never ended with the same sound. The nonwords in the opaque condition were presented seven times in sentences which provided semantic information but no morphological information that could justify the spelling of the final sound (e.g., *Autrefois, les habitants de la campagne se retrouvaient pour la modoit, la fête du village* “In the past, the people of the country met for a /modwa/, the party of the village”). The nonwords in the morphological condition were presented five times, along with two morphologically related nonwords in sentences which provided justification for the spelling of the final sound. For example, in the sentence *La vensoise est une jolie mélodie qui ne se joue qu'avec un vensois*. “The vensoise is a nice melody that can be played only on the vensois,” the presence of a final silent *s* in *vensois* is justified by the pronunciation of this *s* in the morphologically related nonword *vensoise.*

Test questions were printed on the other side of the page so that participants could not see the story when answering the questions. The first test question about each story required participants to select an appropriate title from a list of three. The next three questions were true/false questions. The order of the stories and the nonwords embedded in them were randomized across subjects.

#### Nonword spelling test

For a final forced-choice test, we constructed for each target nonword three phonologically plausible spellings, such as *modoit*, *modois*, and *modoie*. The three spellings differed only in the spelling of the final target sound. Among the incorrect choices, one included a spelling that was used in two other nonwords embedded in the stories. For a correct spelling such as *modoit*, for example, one incorrect spelling was *modois*, ending with the *ois* that is the correct ending in two other nonwords *lagois* and *ridois*. The other incorrect spelling, such as *modoie*, used an ending that was not in any of the nonwords in the stories. The location (left, middle, or right) of the correct and incorrect responses was chosen randomly.

#### General spelling ability

General spelling ability was assessed with the Corbeau standardized spelling subtest (Chevrier-Muller et al., 1997). This test provides a global spelling score which reflects children's ability to produce spellings that are phonologically plausible, even though not necessarily orthographically correct, children's use of word-specific spelling knowledge, and children's correct use of grammatical markers. This test was used to determine whether the spelling ability of the children involved in the study was representative of their expected level.

### Procedure

Children were tested in groups of about 10. They were told that they would receive booklets that included stories along with questions about each. Children were asked to silently read one story and move to the next page to answer questions about it, without rereading the story, then go to the next story, and so on. Children were not told that they would be later asked to remember the spellings from the story and were not told to pay particular attention to the spellings. After this, participants performed a letter cancellation task for 10 min and then were given the standardized spelling test. Finally, about 30 min after having read the stories, children took the final forced-choice spelling test. Children had to circle the correct spelling for each item from the three choices provided.

## Results

Children's answers to the questions indicated that they were fairly successful in reading and understanding the stories. The number of correct responses was reasonably high for the selection of an appropriate title (*M* = 5.29 out of 8, *SD* = 1.54) and the true/false questions (*M* = 19.75 out of 24, *SD* = 2.25). *T* tests using subjects (*t*_1_) and items (*t*_2_) as the random variable showed that performance was significantly above the level expected by chance for both types of questions [*t*_1(27)_ = 9.02; *t*_2(7)_ = 5.36 and *t*_1(27)_ = 27.58; *t*_2(23)_ = 21.36; *p* < 0.001 for both].

The percentage of selection of the correct spellings was higher for items presented in the morphological condition (*M* = 59.82, *SD* = 22.40) than for items presented in the opaque condition (*M* = 45.54, *SD* = 15.67). *T* tests indicated that the selection rate of correct spellings was above chance (33.3%) in the morphological condition [*t*_1(27)_ = 6.26; *t*_2(15)_ = 7.30; *p* < 0.001 for both] and in the opaque condition [*t*_1(27)_ = 4.12, *p* < 0.001; *t*_2(15)_ = 3.48, *p* = 0.003]. The number of selections of correct spellings was submitted to an analysis of variance (ANOVA) with set (A and B) as a between-subject variable and condition (morphological and opaque) as a within-subject variable using subjects as a random variable (*F*_1_) and to an ANOVA with set and condition as between-subjects variables using items as a random variable (*F*_2_). There was a main effect of condition [*F*_1(1, 26)_ = 11.21, *p* = 0.002, η_p_^2^ = 0.30; *F*_2(1, 28)_ = 8.07, *p* = 0.008, η_p_^2^ = 0.22] but no effect of list (*ps* > 0.24) and no interaction (*ps* > 0.68).

## Discussion

In French, as in English and some other languages, the spellings of some words are motivated by those of morphologically related words. Here we asked whether children take advantage of this aspect of the orthography. Several studies have suggested that children begin to do so as early as in first year of formal schooling, based on evidence that they spell words that have morphologically relatives better than words that do not (e.g., Sénéchal, 2000 in French; Treiman et al., 1994 in English). However, most of these previous studies have an important limitation. Even if specific words are balanced for frequency, the observed difference in spelling performance between words with morphological relatives and those without could reflect a difference in the frequency of particular target segments within words. For example the segment *dirt* in *dirty*, which also occurs in *dirty* and *dirtiness*, is more frequent than the segment *dut* in *duty*, which occurs only in *duty*. Deacon and Bryant (eg., 2005a, 2006a,b) attempted to circumvent this potential alternative explanation by having children fill in the missing letters in such items as __ed for *rocked* or by providing clue words such as *payment* to spell items such as *pavement*. However, this method makes the spelling task somewhat artificial.

In the present study, we used a new method to ask whether children use morphologically related words to spell other words and to do so in such a way that positive results cannot be explained by a difference in the frequency of target segments within words. Specifically, we presented the nonwords of interest in stories and then tested children's memory for their spellings. A nonword was presented seven times in a story in the opaque condition. In the morphological condition, the nonword was presented five times in its stem form and another two times in a derived form. If the frequency of occurrence of the nonword itself is the major determinant of children's orthographic learning, then children should spell the nonwords of the opaque condition better than those of the morphological condition. Indeed, previous results show that whole-word frequency is a major determinant of spelling accuracy with real words (e.g., Alegria and Mousty, 1996; Lété et al., 2008) and an important influence on performance in experiments in which children are exposed to novel spellings (Nation et al., 2007). If the presentation of morphologically related words is helpful, however, children may perform better in the morphological condition than the opaque condition even though the nonwords of interest were presented less often in the morphological condition. This is the result that we found. The impact of morphological information, evidently, was strong enough to counteract the whole-word frequency effect. The benefit of morphological relatedness would have probably been larger if items in the morphological and opaque conditions have been matched on the frequency of the whole word, rather than on the frequency of the stem. However, matching items on the frequency of the whole word would not have allowed us to determine whether the difference between the two conditions reflected the use of morphological relatedness or a difference in stem frequency.

Another important characteristic of the procedure used in this study concerns the number of nonwords (16 spread over eight texts) and the interference resulting from the fact that the four target sounds were written with one spelling in two nonwords and with another spelling in two others. We used this procedure to ensure that children acquired word-specific knowledge. For example, children had to learn that the nonword /modw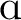/ was spelled with *ois* and not simply that the (only) new word including the final sound /w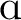/ was spelled *ois.* There is little doubt that this characteristic made our learning situation rather difficult. This may explain why the level of orthographic learning, although above chance, was not very high even in the morphological condition. However, the important result is that children used the principle of root consistency, which states that the roots of words often retain their spelling in related words.

Some tasks involving nonwords, as when children are asked to indicate which of *xihhel* and *xxihel* looks more like a real word (Pacton et al., 2001), do not mirror what normally happens in everyday life. Here, we used nonwords in a task that models the common situation in which people encounter a new word during the course of reading for meaning and later try to spell it. Indeed, much of what people know about spelling is learned this way rather than through explicit teaching (e.g., Treiman and Cassar, 1997; Pacton et al., 2001, 2005; Steffler, 2001). Many studies have embedded nonwords in texts to examine orthographic learning via self-teaching (e.g., Share, 1999, 2004; Nation et al., 2007; Wang et al., 2011). These studies have explored the effects of various variables including number of exposures, whether reading is silent or aloud, and whether words are presented in isolation or in texts (e.g., Nation et al., 2007). The current study is the first to use morphologically related words in such a task and to show that children benefit from morphological relatedness to learn the spelling of new words.

We tested third graders in our study, the first with this new procedure, but future studies could use this paradigm with younger children to examine when they begin to use morphologically related words to learn new spellings. Past work has found that young children's use of morphology in spelling is influenced by the cognitive demands of the tasks (Pacton and Deacon, 2008) and that recognition tasks are easier for young children than production tasks (Totereau et al., 1997). These results suggest that the main finding of our study—that orthographic learning was better in the morphological than the opaque condition—might be seen in younger children with a spelling choice task but not a production task. It would also be valuable to vary the delay between reading and assessment of orthographic learning, as several previous studies have done (e.g., Nation et al., 2007). If morphologically related words serve as a reminder of the correct spelling after a prolonged delay, then performance should decrease more slowly with delay in the morphological condition than in the opaque condition. Although work remains to be done, the present results are important in showing that children use morphology in learning new spellings and that morphological relatedness is important above and beyond frequency of exposure to a specific item.

### Conflict of interest statement

The authors declare that the research was conducted in the absence of any commercial or financial relationships that could be construed as a potential conflict of interest.

## Appendix A

Nonwords embedded in the stories. The target nonwords of the opaque condition are shown in italics, the target nonwords of the morphological condition in bold, and the morphologically related nonwords are underlined.

### Set A

*penin* (/pən), **mansin** (/mãs), mansine (/mãsin/), mansinage (/mãsina\textyogh/)

*pirain* (/pir), **fenain** (/fən), fenaine (/fənεn/), fenainois (/fənεnwa/)

*modoit* (/modwa/), **vensoit** (/vãswa/), vensoite (/vãswat/), vensoitiste (/vãswatist/)

*lagois* (/lagwa/), **ridois** (/ridwa/), ridoison (/ridwazõ/), ridoisine (/ridwazin/)

*pougard* (/pugar/), **coirard** (/kwarar/), coirarde (/kwarard/), coirardage (/kwarardaƷ/)

*togars* (/togar/), **tonvars** (/tvar/), tonvarsine (/tvarsin/), tonvarset (/tvarsε/)

*duband* (/dybã/), **loufand** (/lufã/), loufanderaie (/lufãdərε/), loufandier (/lufãdje/)

*fébant* (/febã/), **roivant** (/rwavã/), roivantier (/rwavãtje/), roivante (/rwavãt/)

### Set B

*penain* (/pən/), **mansain** (/mãs/), mansaine (/mãsεn/), mansainage (/mãsεnaƷ/)

*pirin* (/pir/), **fenin** (/fən/), fenine (/fənin/), feninois (/fəninwa/)

*modois* (/modwa/), **vensois** (/vãswa/), vensoise (/vãswaz/), vensoisiste (/vãswazist/)

*lagoit* (/lagwa/), **ridoit** (/ridwa/), ridoiton (/ridwatõ/), ridoitine (/ridwatin/)

*pougars* (/pugar/), **coirars** (/kwarar/), coirarse (/kwarars/), coirarsage (/kwararsaƷ/)

*togard* (/togar/), **tonvard** (/tvar/), tonvardine (/tvardin/), tonvardet (/tvardε)

*dubant* (/dybã/), **loufant** (/lufã/), loufanteraie (/lufãtərε/), loufantier (/lufãtje/)

*féband* (/febã/), **roivand** (/rwavã/), roivandier (/rwavãdje/), roivande (/rwavãd/)

### Appendix B

Sample story and questions. The target nonwords of the opaque condition are shown in italics, the target nonwords of the morphological condition in bold, and the morphologically related nonwords are underlined. The words were all presented in normal font when the story was presented to participants.

Parmi les instruments de musique, le **vensoit** est peut-être le plus difficile à apprendre. C'est pour cette raison que dans les orchestres, le vensoitiste, le joueur de **vensoit**, est souvent le plus âgé des musiciens. Le **vensoit** est un instrument très particulier qui ressemble à une contrebasse avec plus de cinquante cordes. Pour jouer, il faut les conna^ıtre toutes et savoir utiliser un *fébant* pour les manipuler. Le *fébant* est une petite pince en bois très souple. On tire les cordes du **vensoit** avec le *fébant* pour les faire vibrer. Le *fébant* tord les cordes ce qui donne un son unique qui dure plus ou moins longtemps: tout dépend de l'endroit où le *fébant* est placé sur la corde. Le son de chaque corde vient aussi de l'épaisseur et de la tension des cordes: il y en a des grosses et des fines, des tendues et des moins tendues. La vensoite est une jolie mélodie qui ne se joue qu'avec un **vensoit** et parfois uniquement avec les doigts, c'est-à-dire sans *fébant*. C'est quand on est un très bon musicien qu'on peut jouer sans *fébant*.

1. **Entoure le titre qui convient**
  Un instrument difficile
  Une belle musique
  L'orchestre
2. **Réponds par vrai ou faux**
  L'instrument ressemble à une flûte Vrai / Faux
  Apprendre à jouer est très long Vrai / Faux
  Les cordes sont toutes de la même taille Vrai / Faux

(Among musical instruments, the **vensoit** is probably the most difficult to learn. This is why in orchestras the vensoitiste, the player of **vensoit**, is often the oldest musician. The **vensoit** is a very special instrument that looks like a bass with more than fifty strings. In order to play it, it is necessary to know all the strings and how to use a *fébant* in order to manipulate them. The *fébant* is a little pincer made of wood and is very flexible. One pulls the strings of the **vensoit** with the *fébant* in order to make them vibrate. The *fébant* twists the strings, and this gives a unique sound that is more or less long depending on the position of the *fébant* on the string. The sound of each string depends also of the thickness and the tension of the strings: some are thick and others are thin; some are very tight and others are not tight. The vensoite is a nice melody that can be played only on the **vensoit** and sometimes only with the fingers, that is, without the *fébant*. Only very good musicians can play without a *fébant*).

1. **Circle the best title**
  A difficult instrument
  A nice piece of music
  The orchestra
2. **Answer true or false**
  The instrument looks like a flute True / False
  Learning to play takes a very long time True / False
  The strings are all the same size True / False
